# Blood pressure differences between patients with lacunar and nonlacunar infarcts

**DOI:** 10.1002/brb3.353

**Published:** 2015-06-02

**Authors:** Marianne Altmann, Bente Thommessen, Ole Morten Rønning, Antje S Reichenbach, Brynjar Fure

**Affiliations:** 1 Department of Neurology, Medical Division, Akershus University HospitalLørenskog, Norway; 2 Institute of Clinical Medicine, University of OsloOslo, Norway; 3 Specialist Health Section, The Norwegian Knowledge Centre for the Health ServicesOslo, Norway; 4 Department of Geriatric Medicine, Oslo University HospitalOslo, Norway

**Keywords:** Blood pressure, hypertension, lacunar infarct, lacunar stroke, risk factors

## Abstract

**Background:**

Elevated blood pressure is frequently seen in acute stroke, and patients with lacunar and nonlacunar infarcts may have different underlying mechanisms for increase in blood pressure. The impact of hypertension as a risk factor may also vary. The aims of the present study were to investigate blood pressure in patients presenting with lacunar syndromes but with different anatomical subtypes of stroke, to explore the impact of subtype on blood pressure, and to identify stroke-related factors associated with hypertension.

**Methods:**

Consecutive patients presenting with an acute lacunar syndrome were enrolled. Patients were classified into a lacunar or nonlacunar group based on radiological verified infarcts. Blood pressure was measured. Between-group differences were analyzed by *χ*^2^-test, *t*-test, and Mann–Whitney *U* test, as appropriate. We performed linear regression to analyze the association between blood pressure and lacunar infarct, and multiple linear regression to adjust for other covariates.

**Results:**

One hundred thirteen patients were included. Seventy five percent had lacunar and 25% nonlacunar infarcts. There was no significant difference in clinical severity between the two groups. In the linear regression model, we found a significant association between blood pressure and lacunar infarct. No other factor was significantly associated with blood pressure in the two groups.

**Conclusions:**

Lacunar infarcts may be independently associated with higher blood pressure compared to nonlacunar infarcts with the same clinical severity. Blood pressure differences between different subtypes of stroke may not be related to clinical severity but to the underlying cause of stroke.

## Introduction

Ischemic stroke is anatomically differentiated in lacunar, cortical, and large subcortical infarcts. Lacunar infarcts are located deep in the brain, and are caused by occlusion of a single perforating end artery. Hypertension is the single most important risk factor in all strokes. Traditionally, it was thought that high blood pressure (BP) was strongly associated with lacunar infarct in particular (Fisher 1982; You et al. 1995), but newer studies have shown that risk factors of lacunar stroke do not differ from other stroke subtypes (Boiten et al. 1996; Jackson and Sudlow 2005). Jackson et al. (2010) found no difference between lacunar and nonlacunar stroke regarding occurrence of hypertension.

Hypertension in acute stroke may be a marker of stroke severity but also a physiological response to maintain perfusion in the ischemic penumbra. In 75–80% of patients with acute stroke, blood pressure is transiently elevated with a spontaneous decrease within the following days (Wallace and Levy 1981; Britton et al. 1986; Carlberg et al. 1991; Harper et al. 1994a). The exact mechanism behind this remains unclear. Disturbed cerebral autoregulation (Olsen et al. 1994a), compression of brain regions that regulate the autonomic nervous system, pain, acute sympathetic reaction to the strain and anxiety of a critical illness and hospitalization are all possible mechanisms (Qureshi 2008). A study using 24 h BP monitoring among patients with stroke and hospitalized controls, showed that “white coat hypertension” and hospitalization are unlikely to be the sole factors of high BP (Harper et al. 1994b).

Clinical stroke severity is associated with admission blood pressure, and is correlated with higher blood pressure in all ischemic subgroups with the exception of lacunar stroke (Vemmos et al. 2004a). The underlying mechanisms for the acute elevated BP may be different for lacunar and cortical infarcts, that is, there are differences in the extent of edema and reversible ischemic penumbra, hemorrhagic transformation, and also differences in etiology. The cause as well as the consequence of high BP and BP variations in acute stroke may depend on the subtype of stroke.

The knowledge regarding natural patterns of untreated acute hypertension in different anatomical subtypes of stroke is scarce. We therefore conducted a study to compare blood pressure in patients presenting with clinical lacunar syndromes, but with different radiological subtypes of ischemic stroke, in order to explore the impact of subtype on BP in the acute phase. In addition, we wanted to identify stroke-related factors associated with hypertension in these patients.

## Materials and Methods

Patients presenting with an acute clinical lacunar syndrome who were admitted to the stroke unit of Akershus University Hospital from February 2011 to January 2013 were consecutively included. All patients consented to the study. The methods have been described in detail elsewhere (Altmann et al. 2014).

The diagnosis of a lacunar syndrome was based upon the patients’ history and neurological examination (clinical findings compatible with a lacunar syndrome). Patients who were treated with intravenous thrombolysis were included, even when their symptoms lasted less than 24 h. Exclusion criteria were intracerebral hemorrhage and transient ischemic attack (TIA, symptoms lasting <24 h and no visible infarct on imaging). Patients who had no visible acute infarct on radiological examination were excluded.

The patients underwent standard examination at our stroke unit, including blood samples, electrocardiogram records (ECG), cerebral computed tomography (CT) at admittance and color duplex of precerebral and intracranial arteries. We registered stroke risk factors (e.g. hypertension, smoking, atrial fibrillation, hypercholesterolemia, ischemic heart disease, diabetes, large vessel disease, and previous stroke, or TIA), and defined prestroke hypertension as on-treatment with antihypertensive drugs. Elevated blood pressure was defined as systolic BP > 140. Hypercholesterolemia was defined as on-treatment with lipid-lowering drugs or total cholesterol >5 mmol/L, or low-density lipoprotein cholesterol >3 mmol/L. Findings of symptomatic carotid or middle cerebral artery stenosis ≥50% were registered. Patients underwent magnetic resonance imaging (MRI) with diffusion-weighted images (DWI) within a week after admittance to hospital (Philips Achieva, Royal Philips, Amsterdam, The Netherlands). Due to capacity problems in the MRI scanning, 33 patients underwent only CT scanning. Isolated acute ischemic lesions on DWI or CT were defined as lacunar infarcts if <15 mm and located subcortically or in the brainstem (Wessels et al. 2006), whereas all other acute ischemic lesions were defined as nonlacunar infarcts. All included patients were examined clinically by an experienced stroke neurologist (M.A.). Neurological impairment was assessed by using the 11-items version of the National Institutes of Health Stroke Scale (NIHSS) on day 1 and at discharge (Goldstein and Samsa 1997). Global function was evaluated using the modified Rankin Scale (mRS) at discharge (Rankin 1957; van Swieten et al. 1988).

Blood pressure (BP) registrations included in this study were performed immediately after admission and bedside 6 o’clock in the morning on day 3, and were registered prospectively. BP measurements were performed after standardized protocol, with fully automatic arm blood pressure monitors with the patient in a supine position.

Patients were categorized into two groups based on the size and localization of the infarct, that is, the lacunar infarct group (LI) and the nonlacunar infarct group (NLI).

### Statistical analyses and ethical aspects

Data were analyzed using SPSS version 19 (SPSS Inc., Chicago IL). All significance tests were two-tailed and performed at the 5% level. Continuous variables were expressed as means (±SD) or medians (IQR), and were compared using Student*-t*-test. Normality of continuous variables was assessed by inspecting the histograms. The Mann–Whitney test was used to compare continuous variables showing a non-normal distribution. Categorical variables were compared using the *χ*^2^ test. Linear regression analysis was performed to identify association between blood pressure (at admission and day 3) and lacunar infarct. The regression coefficients are presented as the average difference with 95% confidence intervals (CI) between those with and without lacunar infarct. Covariates in the multiple regression models were: age, gender, hypercholesterolemia, diabetes, large vessel disease, prestroke hypertension, smoking, and NIHSS. We performed backward elimination, removing variables with significance level *P* > 0.05 one by one, to see if any of the other factors were associated with systolic blood pressure day 3, see Table 2007.

Oral and written informed consent was obtained from all included patients. The study was approved by The Regional Committee for Ethics in Medical Research.

## Results

**Figure 1:**
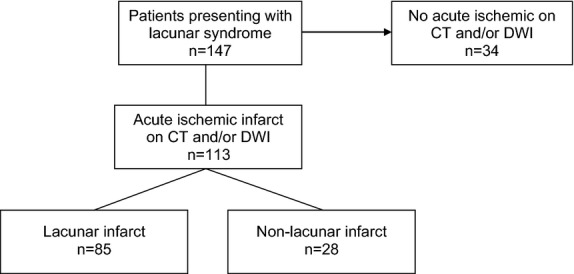
Enrolment diagram.

One hundred forty seven patients presenting with symptoms compatible with a lacunar syndrome were recruited. Thirty three patients underwent only CT scanning, 114 patients both CT and DWI. Thirty four patients had no sign of acute ischemic lesion on CT or DWI and were excluded from the analyses due to uncertainty about stroke subtype, see Fig.1. Eighty five patients (75.2%) had a lacunar infarct (LI), 28 patients (24.8%) had one or more nonlacunar infarcts (NLI). The lacunar infarcts were localized in the basal ganglia (44%), the periventricular white matter (21%), the thalamus (14%), and in the brainstem (21%). The nonlacunar infarcts were localized in the cortex or subcortically, but none were due to occlusion of a major vessel. None of the NLIs located subcortically were lesions consistent with occlusion of a single perforant artery.

The mean age was 70.1 years (SD = 11.5), and 69% were men. The median NIHSS score was 3 (IQR 2–4) at admission and 1 (IQR 0–3) at discharge, whereas the median mRS score was 2 (IQR 1–3) at discharge and median Barthel ADL index (day 2–4) was 20 (IQR 16–20). Of 75.2% of the patients had systolic BP > 140 mm Hg at admission and 54.9% had used antihypertensive medication before admission (prestroke hypertension). There were significantly more patients with systolic BP > 140 among patients with lacunar infarct (LI) than non-lacunar infarct (NLI) on day 3. The systolic blood pressure at day 3 was significantly higher in the LI group than the NLI group (*P* = 0.002). Characteristics and vascular risk factors regarding patients with LI and NLI are presented in Table 2014.

**Table 1:** Characteristics and risk factors.

	Lacunar group *n *=* *85	Nonlacunar group *n *=* *28	*P* value
Age, mean (SD)	69.0 (±11.6)	74.0 (±10.0)	0.068
Males	60 (70.6)	19 (67.9)	0.532
Current smokers	34 (40.0)	5 (17.9)	0.033
Prestroke hypertension^1^	44 (51.8)	18 (64.3)	0.248
Atrial fibrillation	11 (12.9)	6 (21.4)	0.518
Diabetes	19 (22.4)	9 (32.1)	0.298
Statins	30 (35.3)	12 (42.9)	0.473
Large vessel disease^2^	11 (12.9)	6 (21.4)	0.276
Coronary disease^3^	17 (20)	4 (14.3)	0.500
Previous stroke or TIA	14 (16.5)	6 (21.4)	0.551
NIHSS at admission, median, (IQR)	3 (2–4)	2 (1.25–3.75)	0.218
NIHSS at discharge, median, (IQR)	2 (1–3)	1 (0–2)	0.112
Modified Rankin Scale at discharge, median, (IQR)	2 (1–3)	2 (1.25–2)	0.458
Barthel ADL index day 2–4, median, (IQR)	20 (15–20)	20 (17.25–20)	0.738
Systolic blood pressure at admission	183.1 (±31.6)	169.4 (±32.6)	0.066
Diastolic blood pressure at admission	93.1 (±17.7)	87.9 (±15.7)	0.168
Systolic blood pressure day 3	155.5 (±26.5)	139.2 (±21.2)	0.002
Diastolic blood pressure day 3	81.8 (±13.9)	74.0 (±10.7)	0.008

Results are n and (%) unless indicated otherwise.

TIA, transient ischemic attack; ADL, Activities of Daily Living; IQR, Interquartile range; mRS, modified Rankin Scale; NIHSS, National Institutes of Health Stroke Scale; SD, Standard deviation.

1 On-treatment at admission.

2 >50% stenosis in the internal carotid artery or middle cerebral artery.

3 Previous myocardial infarction and/or angina pectoris.

In the linear regression model, there was a significant association between systolic BP (both at admission and at day 3) and lacunar infarct, see Tables 2011 and 1996. Adjusting for covariates, these associations were still significant. There was also a significant association between diastolic blood pressure at day 3 and lacunar infarct (unadjusted, *P* = 0.005, adjusted, *P* = 0.036). None of the other covariates was significantly related to the blood pressure, see Table 2007. Blood pressure was not associated with mRS or NIHSS at discharge (*P* = 0.777 and *P* = 0.887, respectively).

**Table 2:** The association between systolic BP at admission and lacunar infarct, linear regression analysis.

	Unadjusted analysis		Adjusted^1^ analysis	
	Coefficient (95% CI)	*P* value	Coefficient (95% CI)	*P* value
Lacunar infarct	14.1 (0.5–27.6)	0.042	15.1 (0.2–30.0)	0.047

BP, blood pressure; CI, confidence intervals.

1 Adjusted for age, gender, smoking, prestroke hypertension, diabetes, hypercholesterolemia, large vessel disease, and NIHSS.

**Table 3:** The association between systolic BP day 3 and lacunar infarct, linear regression analysis.

	Unadjusted analysis		Adjusted^1^ analysis	
	Coefficient (95% CI)	*P* value	Coefficient (95% CI)	*P* value
Lacunar infarct	16.6 (5.8–27.3)	0.003	16.8 (5.0–28.6)	0.006

BP, blood pressure; CI, confidence intervals.

1 Adjusted for age, gender, smoking, prestroke hypertension, diabetes, hypercholesterolemia, large vessel disease, and NIHSS.

**Table 4:** Factors associated with systolic BP day 3. Multiple regression, backward elimination.

Variables	Bivariate analysis		Multivariate analysis	
	Coefficient (95% CI)	*P*-value	Coefficient (95% CI)	*P*-value
Lacunar infarct	16.6 (5.8 to 27.3)	0.003*	16.2 (5.3 to 27.0)	0.004*
Age	−0.1 (−0.5 to 0.3)	0.555	0.1 (−0.4 to 0.5)	0.685
Male	1.0 (−9.5 to 11.4)	0.855	0.1 (−10.9 to 11.0)	0.989
Prestroke hypertension	−3.9 (−13.6 to 5.8)	0.425	−2.2 (−12.2 to 7.8)	0.668
Smoking	7.8 (−2.3 to 17.9)	0.128	4.6 (−5.4 to 14.7)	0.357
Large vessel disease	−8.2 (−21.6 to 5.3)	0.232	−6.6 (−19.7 to 6.5)	0.318
Hypercholesterolemia	−4.5 (−15.8 to 6.8)	0.431	−2.3 (−13.8 to 9.3)	0.697
Diabetes	5.7 (−5.5 to 16.8)	0.317	7.2 (−3.6 to 17.1)	0.188
NIHSS at admission	−0.364 (−2.5 to 1.8)	0.741	−0.5 (−2.6 to 1.6)	0.639

BP, blood pressure; NIHSS, National Institutes of Health Stroke Scale; CI, confidence intervals.

* Significant association, *P *<* *0.05.

## Discussion

To our knowledge, this is the first study to compare blood pressure in patients with lacunar and nonlacunar infarcts with the same severity of neurological impairments. In this study, patients with acute lacunar infarcts had significantly higher blood pressure on day 3 compared to patients with acute non-lacunar infarcts, regardless of prestroke hypertension. This applies to both diastolic and systolic blood pressure. There was a significant association between blood pressure and lacunar infarct, both at admission and day 3. In contrast to other studies comparing subtypes of stroke, our patient groups had the same severity regardless of subtype. All patients included in this study presented with a clinical lacunar syndrome due to lacunar or nonlacunar infarcts. None of the nonlacunar infarcts were due to occlusion of a major vessel. Accordingly, there was probably a diminutive edema around the infarcts, and therefore a negligible edema effect on the blood pressure. In patients with a major stroke, the blood pressure may rise because of the large volume effect and high intracranial pressure, as a compensatory mechanism, but this was most likely not the case in our study. Smoking was significantly more frequent among patients with lacunar infarct. Earlier studies have documented that smoking is associated with an increased risk of hypertension (Bowman et al. 2007; Thuy et al. 2010). But there was no significant association between smoking and blood pressure in the acute phase in our study.

Few studies have focused on the association between different anatomical subtypes of stroke and different patterns of BP change in acute stroke. Some publications have reported the highest BP levels in patients with lacunar strokes (Semplicini et al. 2003; Toyoda et al. 2006; Meurer et al. 2009), but Rodriguez-Garcia et al. (2005) found higher BP levels from day 1 in patients with nonlacunar strokes compared to lacunar strokes. On the other hand, Vemmos et al. (2004b) found no significant difference in the BP levels between the different etiological subtypes of stroke. In this study, the spontaneous BP variation in acute stroke differed according to subtypes, with a milder drop in cardio embolic strokes compared to end artery small vessel and large vessel atherosclerotic strokes. BP was proportional to the clinical severity of stroke at presentation, which can be explained by the fact that cerebral ischemia might trigger a physiological response, resulting in higher BP.

Our findings of higher blood pressure in patients with lacunar than nonlacunar infarcts correspond to findings in some previous studies (Semplicini et al. 2003; Toyoda et al. 2006). Semplicini et al. (2003) also found that the outcome of stroke was highly associated with the subtype of stroke and initial BP, as lacunar stroke and patients with the highest BP on admission had the best prognosis. In our study of patients with lacunar syndromes, both groups (lacunar and nonlacunar infarcts) had a good clinical outcome with low NIHSS and mRS scores at discharge. BP was not associated with outcome.

Studies evaluating the association between admission blood pressure and outcome has shown conflicting results (Vemmos et al. 2004a,b; Willmot et al. 2004; Ishitsuka et al. 2014). Two studies found that high blood pressure is associated with poor outcome (Willmot et al. 2004; Ishitsuka et al. 2014). They did not look at differences between subtypes of stroke. Kvistad et al. (2013) found an inverse association between BP and stroke severity on admission, where elevated BP was associated with mild stroke, and lack of elevated BP was associated with severe stroke. They assumed that there might be a protective effect of elevated BP. But maybe the high blood pressure in lacunar stroke is a marker of the underlying cause, instead of a protective mechanism? In previous studies exploring the association between the stroke subtype, blood pressure, and outcome, the severity differs between the subtypes. Hence, the question whether differences in admission blood pressure and early change in blood pressure are explained by etiological subtype or severity of neurological deficits is still under debate.

In the present study, three out of four patients had elevated BP at admission, and one out of two used antihypertensive treatment at admission, that is, had prestroke hypertension. There were no significant differences in risk factor profiles between the lacunar and nonlacunar infarct group, except from smoking. Hypertension is the principal risk factor of stroke, but may be even more important in lacunar stroke than in other subgroups. The sustained high blood pressure may be an indication of untreated chronic hypertension. The influence of different subtypes of strokes on blood pressure patterns has not been explored in observational studies or in randomized studies of blood pressure lowering therapies in acute stroke. Recent studies of dynamic cerebral autoregulation have shown bilateral impairment (i.e., impairment of autoregulation in both hemispheres) in patients with lacunar infarct, suggesting chronic pathology of the small vessels (Immink et al. 2005; Guo et al. 2014). In patients with middle cerebral artery territory stroke, there was only ipsilateral impairment of the dynamic cerebral autoregulation. Stroke patients in the highest age groups often suffer large territory infarcts due to cardio embolism. High blood pressure is associated with unfavorable stroke outcome and increased mortality in the elderly (Weiss et al. 2013). Accordingly, future studies of acute stroke and blood pressure should include analyses of stroke subtype as well as severity and age.

Jackson and Sudlow discussed the importance of risk factor-free classifications of stroke subtypes when comparing risk factor profiles between lacunar and nonlacunar subtypes (Jackson and Sudlow 2005). Studies using DWI have documented a low diagnostic accuracy of the lacunar syndrome (Naess et al. 2009; Asdaghi et al. 2011; Altmann et al. 2014). This means that studies without radiologically verified infarcts may have a mix of different subtypes classified as lacunar infarct, which might be a bias. Strengths of our study compared with other studies of blood pressure and subtype were that we conducted a risk factor-free classification of stroke subtypes (i.e., risk factors were not included in the ischemic stroke subtype definition), and that all infarcts were verified by radiological examination. Limitations are the relatively few patients in our study and no continuous monitoring of the blood pressure.

The present study indicates that lacunar infarcts may be independently associated with higher blood pressure compared to nonlacunar infarcts with the same severity of neurological impairments. Blood pressure differences between different subtypes of stroke may not be related to clinical severity but to the underlying cause of stroke. Hypertension in the acute phase of lacunar infarct is not proven to be harmful but is strongly linked to lacunar infarcts as a risk factor. Further studies of blood pressure in the different etiological subgroups of stroke are needed in order to differentiate treatment. We recommend using risk factor-free definitions and ensuring that infarcts are radiologically verified.
